# Phylodynamic and Phylogeographic Analysis of Hepatitis Delta Virus Genotype 3 Isolated in South America

**DOI:** 10.3390/v11110995

**Published:** 2019-10-29

**Authors:** Felipe Souza Nogueira-Lima, Luan Felipo Botelho-Souza, Tárcio Peixoto Roca, Alcione Oliveira dos Santos, Suyane da Costa Oliveira, Jackson Alves da Silva Queiroz, Fabianne Araújo Gomes dos Santos-Alves, Juan Miguel Villalobos Salcedo, Deusilene Souza Vieira

**Affiliations:** 1 Oswaldo Cruz Foundation of Rondônia—FIOCRUZ/RO, Porto Velho RO 76812 245, Rondônia, Brazil; tarcioroca@hotmail.com (T.P.R.); alcione.m@hotmail.com (A.O.d.S.); suyaneoliveira_enf@hotmail.com (S.d.C.O.); queiroz.jas@gmail.com (J.A.d.S.Q.); fabianneag@gmail.com (F.A.G.d.S.-A.); juanitto2001@yahoo.com.br (J.M.V.S.); deusylenebio@hotmail.com (D.S.V.); 2 Research Center in Tropical Medicine of Rondônia -CEPEM/RO, Porto Velho RO 76812 329, Rondônia, Brazil; 3 National Institute of Epidemiology of Western Amazonia—INCT EpiAmO, Porto Velho RO 76812 245, Rondônia, Brazil; 4 Postgraduate Program in Experimental Biology of the Federal University of Rondônia—PGBIOEXP/UNIR, Porto Velho RO 76801 059, Rondônia, Brazil

**Keywords:** hepatitis delta virus, HDV-3, phylodynamics, evolutionary dynamics

## Abstract

The hepatitis delta virus (HDV) is a globally distributed agent, and its genetic variability allows for it to be organized into eight genotypes with different geographic distributions. In South America, genotype 3 (HDV-3) is frequently isolated and responsible for the most severe form of infection. The objective of this study was to evaluate the evolutionary and epidemiological dynamics of HDV-3 over the years and to describe its distribution throughout this continent in an evolutionary perspective. While using Bayesian analysis, with strains being deposited in the Nucleotide database, the most recent common ancestor was dated back to 1964 and phylogenetic analysis indicated that the dispersion may have started in Brazil, spreading to Venezuela and then to Colombia, respectively. Exponential growth in the effective number of infections was observed between the 1950s and 1970s, years after the first report of the presence of HDV on the continent, during the Labrea Black Fever outbreak, which showed that the virus continued to spread, increasing the number of cases decades after the first reports. Subsequently, the analysis showed a decrease in the epidemiological levels of HDV, which was probably due to the implantation of the vaccine against its helper virus, hepatitis B virus, and serological screening methods implemented in the blood banks.

## 1. Introduction

The hepatitis delta virus, which is the sole representative of the *Deltavirus* genus, causes the most severe form of liver infection among all viral hepatitises, with developmental potential for hepatic cirrhosis, hepatocellular carcinoma (HCC), and death [1]. Cirrhotic individuals that were infected with HDV showed a high risk for developing HCC and hepatic decompensation when compared to patients that were infected with hepatitis B virus (HBV) or hepatitis C virus alone [2]. Infection with this virus only occurs when associated with HBV, which makes it a defective and satellite agent, due to the need for an auxiliary virus to provide the envelope/HBsAg during viral replication. This envelope constitutes the outer structure of the HDV virion and it ensures the infectivity of the viral particle [3,4].

In its internal structure is the ribonucleoprotein, which is composed of Delta Antigen (HDAg) molecules that were complexed to a circular ssRNA (−) genome, which is about 1.7 kb in length [5]. Owing to this peculiarity, it has high genetic and evolutionary variability that allows for it to organize itself into eight genotypes (HDV-1 to HDV-8) with extragenotypic differences that can reach values that are close to 40% [6,7,8]. Traditionally, regions with high rates of endemicity are Central and Northern Africa, the Amazon Basin, Eastern Europe and the Mediterranean, the Middle East, and parts of Asia [4,9,10].

HDV-1 is distributed world-wide, being most frequently isolated in the United States, Europe, and the Middle East, as well as in Russia, Africa, Asia, and Brazil. HDV-2 is found in Japan, Taiwan, and Russia. HDV-3, to date, has only been isolated in the Amazon region (Peru, Colombia, Ecuador, and Brazil). HDV-4 is found in Taiwan and Japan. The genotypes HDV-5, HDV-6, HDV-7, and HDV-8 are found in Africa. In South America, only HDV-1 and HDV-3 genotypes have been frequently isolated. However, there are isolated case reports of the presence of HDV-8 in the countryside of the state of Maranhão, Brazil [4,11,12].

In Brazil, 75% of the cases of hepatitis delta between 1999 and 2017 occurred in the northern region of the country, which is an area that partially comprises the Western Amazon [13]. Molecular studies have demonstrated that the HDV-3 variant is most prevalently isolated in this region, both in non-indigenous [14,15] and indigenous populations [16,17,18]. This genotype is apparently related to more aggressive HDV infection [19,20,21,22].

Although the virus was discovered in the late 1970s [23], there are reports that this agent was present in the Amazon region before this period, being involved in outbreaks, such as “hepatitis of the Sierra Nevada de Santa Marta” (Colombia), and in cases of fulminant hepatitis during an outbreak in the southern region of the state of Amazonas (Brazil), denominated Labrea Black Fever [24]. In light of these facts, the objective of this study was to determine the evolutionary dynamics of HDV-3 strains that were isolated in South America, elucidating the behavior of the genetic diversity of the virus over the years, estimating the time to the Most Recent Common Ancestor (tMRCA) and to associate the findings with the environment and natural history of the region.

## 2. Materials and Methods

### 2.1. Study Population

The sequences that were used in the study were obtained through systematized searches in the Nucleotide database—a collection of sequences from various sources, including Genbank (available at: https://www.ncbi.nlm.nih.gov/nucleotide/), which is currently associated with the National Center for Biotechnology Information (NCBI). Using as a descriptor in the searches “hepatitis delta virus genotype 3”, 44 sequences were selected that contained information regarding collection date and the country of isolation. The sequences that were collected were isolated between 1978 and 2011, in Brazil, Colombia, and Venezuela (Table 1).

The analysis was performed from a region that contained 319 bp, herein referred to as R0, which was located at positions NT 917 through 1241 of the HDV genome (position determined from local alignment with the reference sequence for HDV: NC_001653.2, including gaps). This region partially comprises the HDAg gene encoding, including the additional 19 amino acids in the long-form (L-HDAg) C-terminal portion, a highly variable and specific sequence for each genotype that represents the Viral Assembly Signal (VAS) [4]. This region of the complete genome has been used in previous phylogenetic studies [8,15,19,22,25,26], because it allows for more similar phylogenetic trees results than those that are based on the whole genome [8,19,26].

### 2.2. Alignment and Determination of Evolutionary Model

The alignment was performed while using the Clustal W algorithm in MEGA7 software (Molecular Evolutionary Genetic Analysis, [30]). An estimation of the most appropriate substitution model was performed in MEGA7 itself, while using the Model Analyses (ML) tool, through the Automatic (Neighbor-joining tree) tree, showing that the most suitable model for the data set was Tamura three-parameter with Gamma distribution (T92 + G), which was chosen among the others because it has the lowest Bayesian Information Criterion (BIC) value (4187,2987).

### 2.3. Temporal Signal Estimation

To perform Bayesian phylogenetic inference based on a molecular clock, there must be sufficient genetic change between the sampling times of the taxa to reconstruct a statistical relationship between genetic divergence and time [31]. To examine this relationship, an analysis was performed to verify the existence of a temporal signal in the study data set. This analysis was done while using TempEst v.1.5.3 software, through a “non-clock” phylogenetic tree that was obtained while using IQtree-1.6.10. software.

### 2.4. Bayesian Analysis

In the software BEAUti v.1.10.1., the collection date for the Bayesian inference was included to reconstruct the dynamic and evolutionary past of the virus. The uncorrelated relaxed clock was defined by allowing each branch of the phylogenetic tree to have a different evolutionary rate than the others. Tamura-Nei (TN93) (BIC: 4209,630) was used as an evolutionary model, since T92 is not included among this software’s models.

The effective sample size (ESS) of parameters post-run of Markovian chains is indicated to be greater than 200. The Bayesian SkyGrid coalescent model was used. 50 Tree Prior parameters were defined, with a final time point of 100 years before the most recent sampling, estimating one population size every two years. The length of the Monte Carlo Markov Chain (MCMC) was set to 5 × 10^7^, with data collection every 5000, which thus creates 10,000 samples by running Markov chains through BEAST v.1.10.1 software, which was sufficient for the convergence of parameters. The analyses were performed in duplicate and were combined while using LogCombiner v.1.8.4 software.

Bayesian reconstruction and the determination of tMRCA were inferred while using Tracer v.1.7.1 software. The phylogenetic tree was summarized and a consensus tree was created using TreeAnotator v.1.10.1., which in turn selects a single “target” tree and records it with the summary information of the entire sample of trees (10,000), excluding 10% of the samples as burn-in, creating a Maximum Clade Credibility (MCC) tree. The summarized information includes the average node ages, along with the Highest Posterior Density (HPD) intervals, which are calculated for each node or the clade of the consensus tree. The consensus tree was visualized and customized while using FigTree v.1.4.3.

### 2.5. Spatial Phylogenetic Reconstruction of the Evolutionary Dynamics

A phylogeographic analysis was performed to infer a dispersion profile from the cartographic point of view. The methodology used resembles that used in the reconstruction of evolutionary dynamics, with small exceptions. The data that refer to the location coordinates of the taxa were implemented during the execution of BEAUti v.1.10.4. The model used was selected from tests and observation of the ESS value to evaluate the convergence of the parameters. Thus, the Cauchy Relaxed Random Walk model was selected for phylogeographic diffusion in continuous space, with an MCMC chain length of 10 × 10^8^ and data collection every 10,000 steps. The results of the analysis were summarized while using TreeAnotator v.1.8.4. for building an MCC tree. This, in turn, was used to plot the diffusion results on the map through SPREAD v.1.0.7 software and the result was viewed through Google Earth software. Specific isolation local of sequence were obtained by reading the studies that gave rise to the deposition. Only the sequence KC590319.1 had no specific isolation local defined (only Brazil was informed as the place of origin), however the phylogenetic analysis allowed for observing that it was similar to those that were isolated in the State of Amazonas, Brazil. Therefore, this state was defined as the origin place of this strain. The coordinates used were obtained by searching in Google Earth (Table 2).

## 3. Results and Discussion

**Figure 1:**
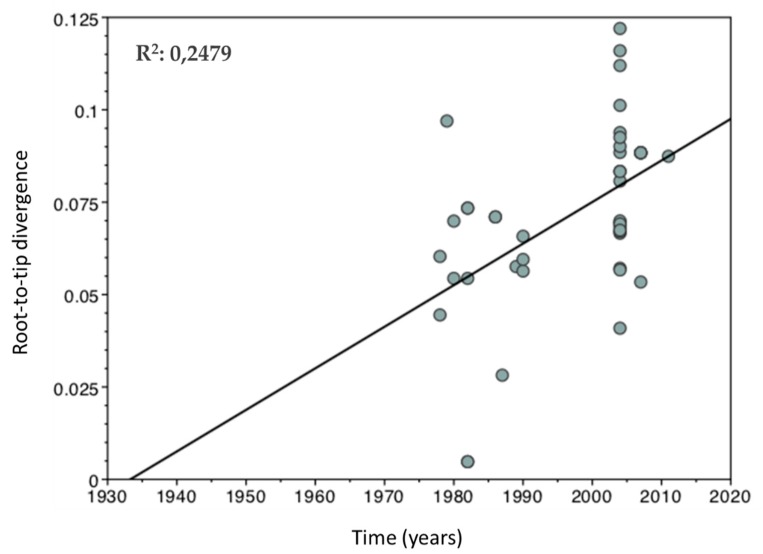
Temporal signal linear regression graph. The graph shows the positive correlation between genetic diversity from root-to-tip (*y*-axis) and time (*x*-axis). This effect on the relationship of these variables shows the existence of a temporal signal in the data set that makes it sufficient to perform molecular clock analysis.

It was possible to observe a linear regression curve that shows a positive correlation between genetic divergence and sampling time by exploring the existence of the temporal signal in the study dataset (Figure 1). This correlation pattern points to the conclusion that the alignment contains enough temporal information to justify a molecular clock approach [31]. Although the temporal signal level is low, as evidenced by the R^2^ value (0.24), this parameter should not be used to test the statistical significance of the regression, because the individual data points are not independently distributed and are instead partially correlated due to shared phylogenetic ancestry [31]. Temporal signal secondary analyses were performed, including the full-length genome and R0 region of 55 HDV-1 sequences deposited in the Nucleotide database and exhibited similar temporal signal levels [32] (data not shown).

**Figure 2:**
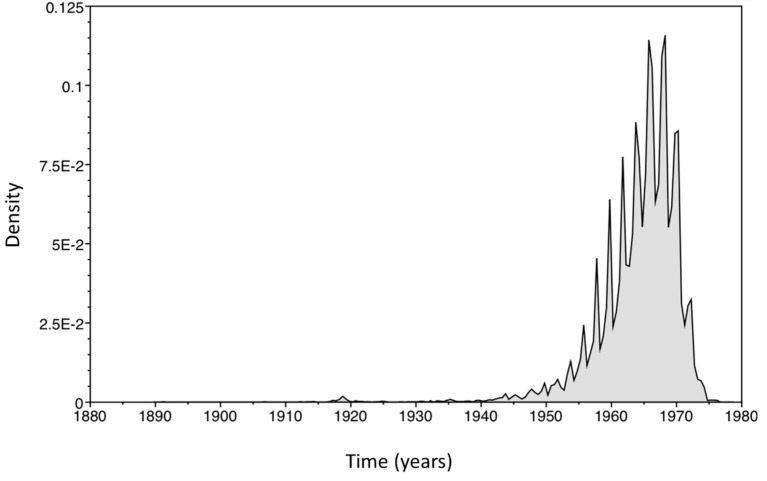
Histogram of age estimates for the tree root. The graph shows the relationship of tree root age estimates (*x* axis) according to the probability frequency range (*y* axis) ranging from 0 to 12.5%. The 95% Highest Posterior Density (HPD) interval presents data ranging from 1952 to 1972. The graph was generated in Tracer v.1.7.1 software.

Evolutionary dynamics analysis being performed in isolated sequences from different regions of South America showed the most recent common ancestor time, dating to 47 years prior to the most recent sample, estimated in 1964 according to the mean of the data (95% of HPD between 1952 and 1972) (Figure 2).

**Figure 3:**
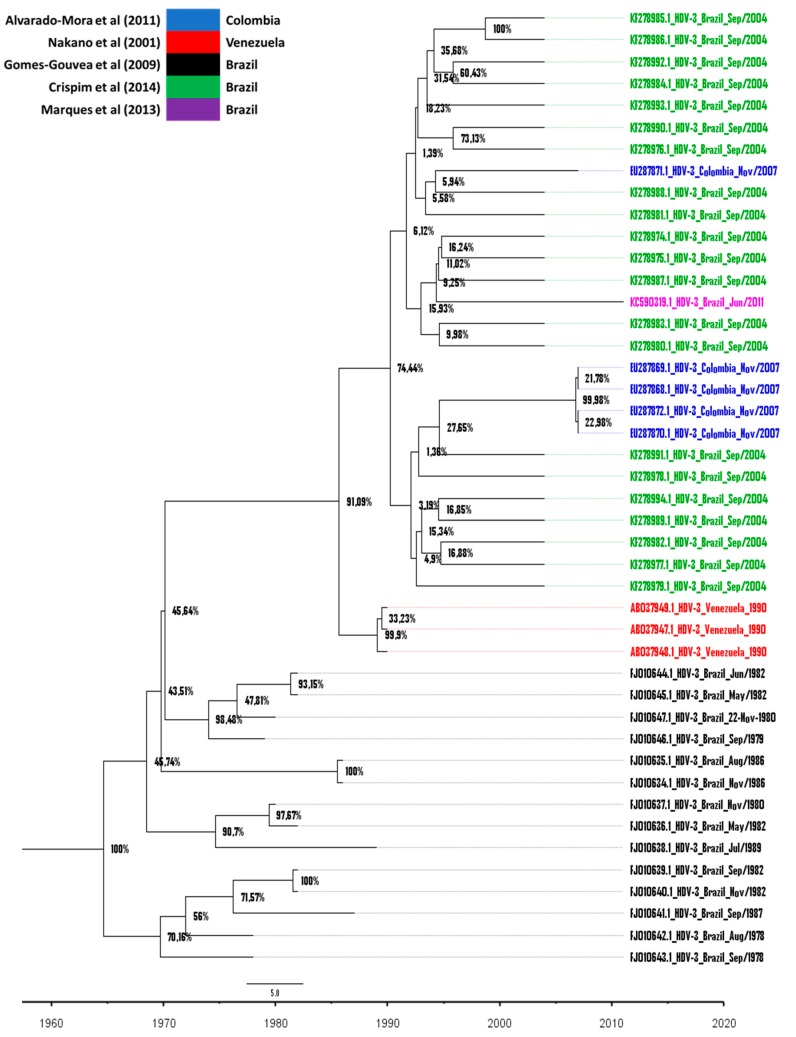
Bayesian phylogenetic tree. In the maximum clade credibility (MCC) tree generated, the phylogenetic relationship was estimated by Bayesian analysis among 44 strains of HDV-3 isolated in South America from 1978 to 2011. Red color taxa correspond to sequences from [28]; blue corresponds to those from [24]; green to those from [27]; black to those from [22] and purple to that from [29] (Table 1). In each node the posterior probability rate is shown as percentage data and in the lower part, the time in years is displayed. The time to the Most Recent Common Ancestor (tMRCA) dating back to 1964 is demonstrated for this tree root.

In the phylogenetic tree constructed (Figure 3), it was possible to observe that the HDV sequences that were isolated in the state of Amazonas (Brazil) [27] have a common ancestor with sequences isolated in Colombia [24] dating around the year 1990 (posterior probability of 74.4%). This grouping shares an ancestor dating five years earlier, with three sequences from Venezuela [28]. These Venezuelan HDV strains were isolated from Yucpa indigenous patients from three distinct, but closely located, villages and lined up in a single cluster, which suggests that a single strain of HDV caused dissemination in this population.

In the early 1980s, the Yucpa Amerindians were affected by a devastating epidemic of HDV [28]. In a 1992 study that involved 216 Yucpa Indian patients with HBV under observation between 1983 and 1988, 74 were initially positive for HDV infection and another 35 contracted the infection during the study, which showed evidence of a high prevalence of the virus in this population [33]. Although the years between the results of the Bayesian analysis are not accurately related to prevalence studies, it is worth mentioning that, in the generated MCC tree, the nodes are located according to the average age estimation results in 95% HPD intervals of these same nodes. This range of probabilities varies, the node in question varied between 1975 and 1989, and the mean of data estimated this node as being the year 1885.

The phylogenetic analysis allowed for us to observe that this cluster of HDV strains from Venezuela, Colombia, and Brazil still share ancestors between 1963 and 1970, with HDV strains being isolated from patients who presented fulminant hepatitis and died, deposited by [14], showing that the spread of HDV occurred in this direction: starting in Brazil, spreading to Venezuela, and then to Colombia. However, there is no relation of heterochronology between the sequences of Venezuela and Colombia, which were collected in 1990 and 2007, respectively. This lack of disparity between the intragroup isolation dates does not allow for us to conclude that HDV did, in fact, spread in this way. In addition, Bolivia and Peru are countries that report HDV isolates, but they do not have collection dates that are specified for the deposited sequences.

**Figure 4:**
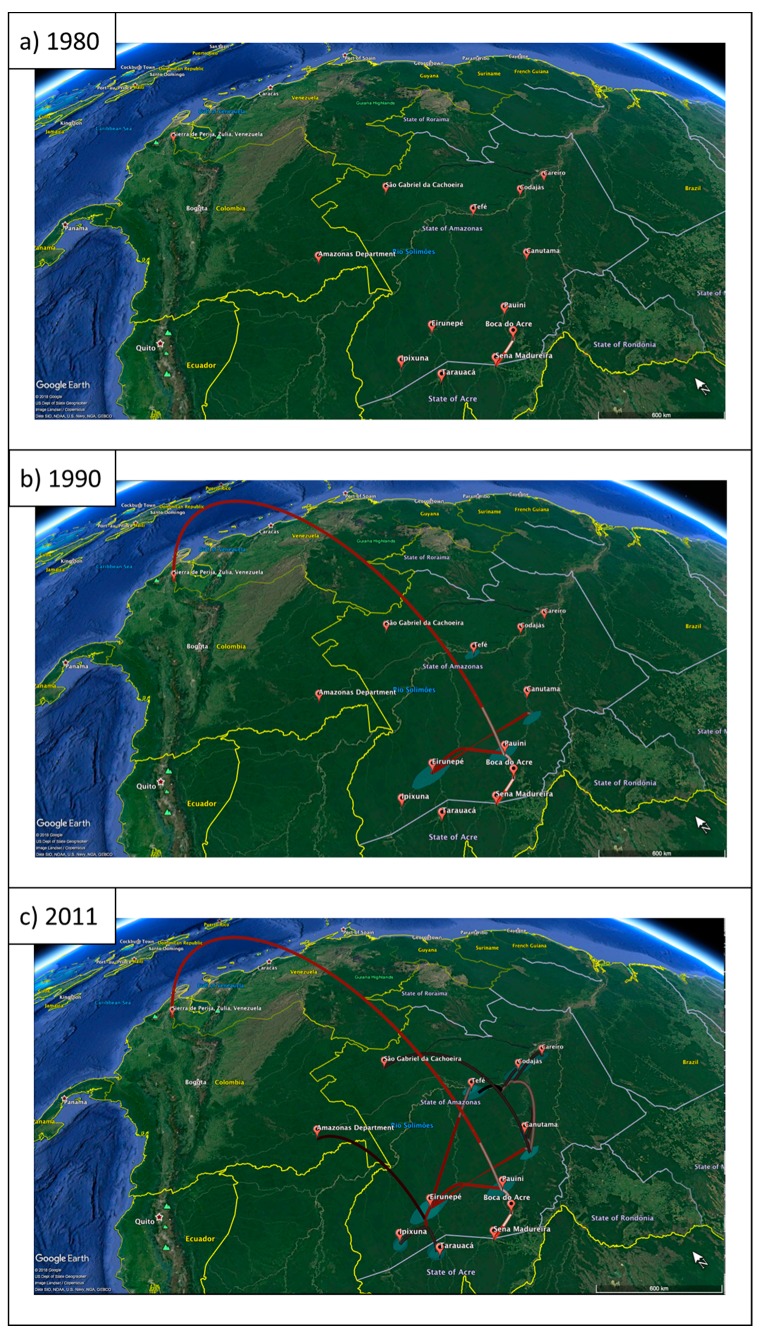
Phylogeographic dispersion of HDV-3 in South America. The spatial phylogenetic reconstruction of the evolutionary dynamics traced in the study is shown in the cartographic plane referring to the region of study. The dispersion lines are indicated according to a time-related color gradient, where red refers to the minimum time and black to the most recent time. Polygons in the plane represent the posterior support of the node, which also accompany a color gradient, ranging from black (lightly supported node) to light blue (well supported node).

According to the phylogeographic analysis that was performed in the study, in 1980, the first possible phylogenetic relationships of HDV-3 are detectable, which quite possibly shows that the dispersion started in the region of Boca do Acre (Amazonas, Brazil) and then arrived at Sena Madureira (Acre, Brazil) (Figure 4A). In the following decade, the Yucpa amerindians of the northwestern region of Venezuela experienced the devastating epidemic of HDV and, according to the results, most probably the strain that caused the HDV infections in this population arose from a focus of dissemination in the municipality of Pauini (Amazonas, Brazil). Given the distance between the points, it is likely that the dissemination occurred through a traveling individual. In this same decade, HDV-3 continued to spread through other points in this region (Figure 4B).

In the last year of the limit of the analysis, 2011, it is already possible to observe that this genotype was even more dispersed in the Amazon region, in the East, West, and North directions. A new focus of dissemination was formed, specifically in the vicinity of the municipality of Eirunepé (Amazonas, Brazil), which, in addition to originating strains that were directed to other municipalities of the same state (Tefé and Ipixuna), also originated those that were directed to the state of Acre, in Tarauacá. The strains of Colombia also originated from ancestors that were linked to the Eirunepé region and arrived in the country causing fulminant hepatitis in patients who were invited to participate in the Alvarado-Mora et al. (2011) [24] study (Figure 4C).

**Figure 5:**
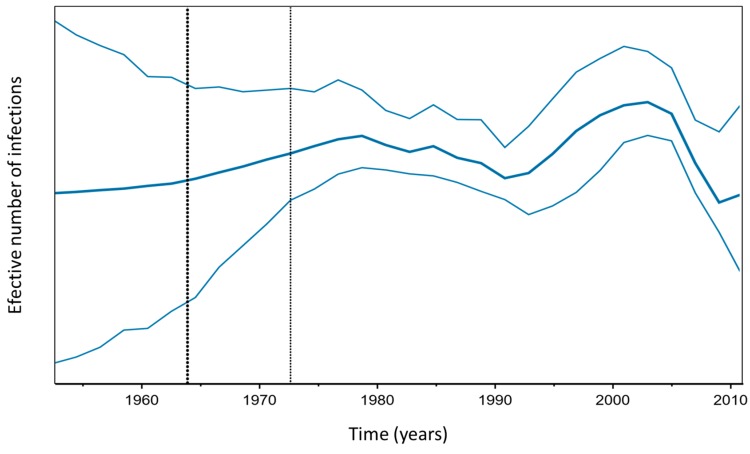
Skygrid reconstruction. In the graph, the relationship between the axes shows the equivalent to the effective number of infections (*y*-axis) and the chronological time expressed in years (*x*-axis). The thick blue central horizontal line indicates the mean, and the fine lines indicate the 95% HPD interval. The first vertical dashed line indicates the mean root age, or tMRCA (1963), and the second dashed line indicates the highest value of this same variable (1972). These estimates were obtained using multiple alignment with 44 HDV sequences that were isolated at different sites in South America at different points in time.

SkyGrid reconstruction analysis (Figure 5) showed slow exponential growth that began in 1952 (tMRCA minimum value) and continued through the mid-1980s. A study from 2011 using only HDV-3 sequences that were isolated in Brazil, Colombia, Venezuela, and Peru, found exponential growth between the 1950s and 1970s, through analysis with the Bayesian Skyline Plot model [24] and showed a brief similarity between the results, when the geographic origin of the analyzed strains was considered.

However, the Bayesian SkyGrid coalescent model that was used for population dynamics inference in the present study presents a more reliable and accurate approach. Presented to the scientific community two years later, in 2013, it has been shown to be more efficient in recovering true population trajectories and it has produced a considerable increase in the accuracy of tMRCA determination [34]. This improvement appears to be especially prominent in multi-loci datasets, such as the analyzed region of the HDV genome, which, in addition to partially comprising the HDAg encoding gene, has nucleotides that correspond to L-HDAg and also a non-coding region. This observation is supported when 95% HPD of the tMRCA value determined by [24] varied by 153 years (1821–1974), while that of the current study varies by only 20 years (1952–1972).

Acclivity in genetic diversity/time relationships can be interpreted as an increase in the effective number of infections over time, and the reverse interpretation is possible when a decline occurs on the graph. This association is only possible when the genomic changes of the analyzed organism do not undergo some strong influence of natural selection [35], as occurs, for example, in the cases of resistance mutations in the genome of HBV and HIV, which arise when these viruses are exposed to drugs whose targets are specific to viral replication activity.

It was stated that HDV was related to old cases of hepatitis on the South American continent. One report that is most likely associated with this virus is known as “Sierra Nevada de Santa Marta” hepatitis, an outbreak that occurred in Colombia [24]. In 1985, 60% of HBsAg-reactive individuals from a village in northern Colombia who had clinical profiles that were consistent with this outbreak were also reactive for anti-HDV [36]. In the following year, a study was performed with 81 patients who presented this hepatitis clinical profile and died, 70% of whom presented the Delta Antigen through autopsy and viscerotomy analyses [37].

The exponential growth between 1952 and 1980 indicates that there was an exorbitant increase in the epidemiological rates of HDV infection during this period. Some years before, precisely in 1938, the presence of HDV in South America was confirmed through histological analysis of hepatic viscerotomy samples and the serology of patients who presented a liver disease, known as Labrea Black Fever, in the southern region of the State of Amazonas, Brazil [38,39]. This outbreak was characterized by severe fast course hepatitis, which resulted in liver failure and death [40]. There are reports of a disease that decimated entire families and affected so-called rubber soldiers and family members, receiving various denominations, including Lábrea Black Fever and Lábrea hepatitis [41], which was later associated with HDV infection. Thus, it is possible to assume that the rate of HDV infection continued to increase among these populations decades after initial reports, until it experienced a stationary period and decline.

The two oldest HDV cases date back to 1938 and 1940, and they were confirmed by histological analysis and Delta antigen detection from patients from the Boca do Acre region of Brazil [38,39]. Dias & Coura (1985) [39] also concluded, histologically, the diagnosis of Lábrea hepatitis in samples of Colombian and Peruvian patients; however there was no detection of the Delta antigen [39]. Currently, it is known that this clinical condition also presents other possible etiologies [40], which makes it impossible to sustain that these diseases were related to HDV. Even if they were, it is not possible to say whether or not the infection was contracted in the country of origin, or whether those patients were residents of Brazil. Therefore, while considering the evolutionary dispersion profile that were observed through the temporal phylogenetic tree (Figure 3) and the order of reports of the presence of HDV in South America—Brazil in 1938, 1940, and 1978 [22,38,39], Venezuela in the mid-1980s [28] and Colombia in 1985 and 1986 [36,37]—it is plausible to conclude that the spread of HDV-3 truly began in Brazil.

In September 1989, vaccination against HBV was implemented in the Amazon region and in 1993 the minimum age for immunization was decreased [17,24]. When considering the fact that HDV infection is only possible when associated with HBV, one would expect the relative number of infections to decrease after this event. However, Bayesian analysis showed that the first decline in the genetic diversity/time relationship occurred only after the year 2000. Seven years after the introduction of the vaccine, a decrease in the prevalence rate of HDV was reported in areas that are considered to be hyperendemic in the Amazon region, from 32% in individuals reactive for HBsAg to 22.2% in individuals with reactivity to any marker of hepatitis B [42,43].

A recent study reported successive reductions in the prevalence rate of HDV over time in South America. In the 1980s, the value of this variable was determined to be 40.29% for HBsAg reactive patients and it decreased to 28.01% by the end of the decade, and then to a moderate endemic level in 2000 (13.58%). It also continued to exponentially decline to values that were close to those of the rest of the world in 2010 (6.97%) [44].

Most of the strains that were used in the analysis were isolated in Brazil. Epidemiological data from this country show that there was a recent fall in the number of registered cases of hepatitis Delta from 359 cases in 2013, to 159 in 2017, with a decrease of 55.72% (mean drop of 13.93% per year). Between 1999 and 2017, 75% of registered HDV infections occurred in the northern region of Brazil [13], which, in addition to partially comprising the Western Amazon Basin, is relatively geographically close to Venezuela and Colombia. Bayesian analysis of HDV evolution presented results that corroborate HDV prevalence studies at this study site from a more specific point of view, which observes how much the viral genome has been modified over time, estimating a field of probabilities that reflect the genetic diversity that is related to the time of the epidemiological behavior of the pathogen. In an indirect way, the study suggests that the result of immunization against HBV and the screening of transfusion materials in blood centers was determinant for the fall in HDV epidemiological levels.

## 4. Conclusions

The decline in the genetic diversity of HDV in the new millennium may be a direct consequence of the implementation of the HBV vaccine and of more efficient screening methods that are directed at transfusion materials in blood banks, since the blood pathway is a strong form of HBV transmission, and consequently HDV. These considerations regarding changes in the genome of the virus over time are sustained by observations in the established epidemiological rates in previous studies that have shown successive declines in the number of HDV cases. However, HDV infection is still a worrisome health problem, especially in endemic regions, which causes the development of fulminant hepatitis and chronic hepatitis, which can progress to liver cirrhosis, hepatocellular carcinoma, and death.

Regarding the evolutionary history, although the most recent ancestor of the strains analyzed was dated back to the 1960s, previous studies have reported the presence of this virus in South America about 30 years earlier, which indicates that infection by this virus has been a serious problem for almost a century, affecting both isolated Amerindian populations and the general population. The evolutionary profile and chronology of HDV reports in South America indicate a possible geographical origin of the virus in Brazil. However, it is recommended to consider that the Bayesian analysis only showed viral epidemiological behavior after 1952 and there is no way to determine how the spread of the virus occurred during previous periods. Therefore, it is possible that there is another origin, since the first confirmed report was of patients with Labrea Black Fever, and these cases began to be reported only after 1926, presuming then that this is the period of arrival of the virus in the Brazil, or a possible founder effect, which may or may not be associated with indigenous populations.

## Figures and Tables

**Table 1:** Information on the sequences that were used in the study.

Access number	Country of Isolation	Collection date	Clinical information	Author
* KF278974.1 to KF278994.1	Brazil	Sep/2004	CH	[27]
* EU287868.1 to EU287872.1	Colombia	Nov/2007	FH	[24]
* AB037947.1 to AB037949.1	Venezuela	1990	FH	[28]
FJ010634.1	Brazil	Nov/1986	FH+D	[22]
FJ010635.1	Brazil	Nov/1986	FH+D	[22]
FJ010636.1	Brazil	May/1982	FH+D	[22]
FJ010637.1	Brazil	Nov/1980	FH+D	[22]
FJ010638.1	Brazil	Jul/1989	FH+D	[22]
FJ010639.1	Brazil	Sep/1982	FH+D	[22]
FJ010640.1	Brazil	Nov/1982	FH+D	[22]
FJ010641.1	Brazil	Sep/1987	FH+D	[22]
FJ010642.1	Brazil	Aug/1978	FH+D	[22]
FJ010643.1	Brazil	Sep/1978	FH+D	[22]
FJ010644.1	Brazil	Jun/1982	FH+D	[22]
FJ010645.1	Brazil	May/1982	FH+D	[22]
FJ010646.1	Brazil	Sep/1979	FH+D	[22]
FJ010647.1	Brazil	Nov/1980	FH+D	[22]
KC590319.1	Brazil	Jun/2011	NA	[29]

* Sequences are grouped based on sampling dates, place of isolation, clinical status and depositing authors. CH: Chronic hepatitis; FH: Fulminant hepatitis; D: Death; NA: Not available.

**Table 2:** Information on place of origin and coordinates used.

Sequence	Latitude	Longitude	Place of isolation
KF278994.1_HDV-3_Brazil_Sep/2004	−3.368333	−64.719167	Tefé (Amazonas, Brazil)
KF278993.1_HDV-3_Brazil_Sep/2004	−7.260000	−64.799167	Labrea (Amazonas, Brazil)
KF278992.1_HDV-3_Brazil_Sep/2004	−0.130278	−67.089167	S.Gabriel da Cachoeira (Amazonas, Brazil)
KF278991.1_HDV-3_Brazil_Sep/2004	−6.660278	−69.874444	Eirunepe (Amazonas, Brazil)
KF278990.1_HDV-3_Brazil_Sep/2004	−5.628333	−63.183611	Tapaua (Amazonas, Brazil)
KF278989.1_HDV-3_Brazil_Sep/2004	−3.368333	−64.719167	Tefe (Amazonas, Brazil)
KF278988.1_HDV-3_Brazil_Sep/2004	−7.260000	−64.799167	Labrea (Amazonas, Brazil)
KF278987.1_HDV-3_Brazil_Sep/2004	−3.837222	−62.057500	Codajas (Amazonas, Brazil)
KF278986.1_HDV-3_Brazil_Sep/2004	−6.660278	−69.874444	Eirunepe (Amazonas, Brazil)
KF278985.1_HDV-3_Brazil_Sep/2004	−6.660278	−69.874444	Eirunepe (Amazonas, Brazil
KF278984.1_HDV-3_Brazil_Sep/2004	−7.260000	−64.799167	Labrea (Amazonas, Brazil)
KF278983.1_HDV-3_Brazil_Sep/2004	−7.260000	−64.799167	Labrea (Amazonas, Brazil)
KF278982.1_HDV-3_Brazil_Sep/2004	−7.714444	−66.976389	Pauini (Amazonas, Brazil)
KF278981.1_HDV-3_Brazil_Sep/2004	−7.051389	−71.695556	Ipixuna (Amazonas, Brazil)
KF278980.1_HDV-3_Brazil_Sep/2004	−3.812222	−60.345556	Careiro (Amazonas, Brazil)
KF278979.1_HDV-3_Brazil_Sep/2004	−6.660278	−69.874444	Eirunepe (Amazonas, Brazil)
KF278978.1_HDV-3_Brazil_Sep/2004	−8.135556	−70.765000	Tarauaca (Acre, Brazil)
KF278977.1_HDV-3_Brazil_Sep/2004	−6.660278	−69.874444	Eirunepe (Amazonas, Brazil)
KF278976.1_HDV-3_Brazil_Sep/2004	−6.533889	−64.383056	Canutama (Amazonas, Brazil)
KF278975.1_HDV-3_Brazil_Sep/2004	−6.660278	−69.874444	Eirunepe (Amazonas, Brazil)
KF278974.1_HDV-3_Brazil_Sep/2004	−6.660278	−69.874444	Eirunepe (Amazonas, Brazil)
EU287872.1_HDV-3_Colombia_Nov/2007	−1.416944	−71.577778	Amazonas/Colombia
EU287871.1_HDV-3_Colombia_Nov/2007	−1.416944	−71.577778	Amazonas/Colombia
EU287869.1_HDV-3_Colombia_Nov/2007	−1.416944	−71.577778	Amazonas/Colombia
EU287870.1_HDV-3_Colombia_Nov/2007	−1.416944	−71.577778	Amazonas/Colombia
EU287868.1_HDV-3_Colombia_Nov/2007	−1.416944	−71.577778	Amazonas/Colombia
FJ010647.1_HDV-3_Brazil_22-Nov-1980	−8.740556	−67.384167	Boca do Acre (Amazonas, Brazil)
FJ010646.1_HDV-3_Brazil_Sep/1979	−8.740556	−67.384167	Boca do Acre (Amazonas, Brazil)
FJ010645.1_HDV-3_Brazil_May/1982	−8.740556	−67.384167	Boca do Acre (Amazonas, Brazil)
FJ010644.1_HDV-3_Brazil_Jun/1982	−8.740556	−67.384167	Boca do Acre (Amazonas, Brazil)
FJ010643.1_HDV-3_Brazil_Sep/1978	−906721	−68.6577	Sena Madueira (Acre, Brazil)
FJ010642.1_HDV-3_Brazil_Aug/1978	−906721	−68.6577	Sena Madueira (Acre, Brazil)
FJ010641.1_HDV-3_Brazil_Sep/1987	−7.714444	−66.976389	Pauini (Amazonas, Brazil)
FJ010640.1_HDV-3_Brazil_Nov/1982	−8.740556	−67.384167	Boca do Acre (Amazonas, Brazil)
FJ010639.1_HDV-3_Brazil_Sep/1982	−8.740556	−67.384167	Boca do Acre (Amazonas, Brazil)
FJ010638.1_HDV-3_Brazil_Jul/1989	−8.740556	−67.384167	Boca do Acre (Amazonas, Brazil)
FJ010637.1_HDV-3_Brazil_Nov/1980	−8.740556	−67.384167	Boca do Acre (Amazonas, Brazil)
FJ010636.1_HDV-3_Brazil_May/1982	−8.740556	−67.384167	Boca do Acre (Amazonas, Brazil)
FJ010635.1_HDV-3_Brazil_Aug/1986	−7.714444	−66.976389	Pauini (Amazonas, Brazil)
FJ010634.1_HDV-3_Brazil_Nov/1986	−7.714444	−66.976389	Pauini (Amazonas, Brazil)
KC590319.1_HDV-3_Brazil_Jun/2011	−3.750000	−64.500000	Amazonas/Brazil
AB037948.1_HDV-3_Venezuela_1990	10.038056	−73.011944	Sierra De Perija (Zulia, Venezuela)
AB037947.1_HDV-3_Venezuela_1990	10.038056	−73.011944	Sierra De Perija (Zulia, Venezuela)
AB037949.1_HDV-3_Venezuela_1990	10.038056	−73.011944	Sierra De Perija (Zulia, Venezuela)

## References

[1] Cunha C., Tavanez J.P., Gudima S. (2015). Hepatitis delta virus: A fascinating and neglected pathogen. World J. Virol..

[2] Romeo R., Facchetti F., Perbellini R., Galmozzi E., Petruzziello A., Di Capua L., Sabatino R., Botti G., Loquercio G., Pecheur E.I. (2018). Hepatitis delta virus and hepatocellular carcinoma: An update. Epidemiol. Infect..

[3] Giersch K., Dandri M. (2015). Hepatitis B and Delta Virus: Advances on Studies about Interactions between the Two Viruses and the Infected Hepatocyte. J. Clin. Transl. Hepatol..

[4] Botelho-Souza L.F., Vasconcelos M.P.A., dos Santos A.O., Salcedo J.M.V., Vieira D.S. (2017). Hepatitis delta: Virological and clinical aspects. Virol. J..

[5] Lempp F.A., Urban S. (2017). Hepatitis Delta Virus: Replication Strategy and Upcoming Therapeutic Options for a Neglected Human Pathogen. Viruses.

[6] Taylor J.M. (2014). Host RNA circles and the origin of hepatitis delta virus. World J. Gastroenterol..

[7] Miao Z., Zhang S., Ma Z., Hakim M.S., Wang W., Peppelenbosch M.P., Pan Q. (2019). Recombinant identification, molecular classification and proposed reference genomes for hepatitis delta virus. J. Viral Hepat..

[8] Le Gal F., Brichler S., Drugan T., Alloui C., Roulot D., Pawlotsky J.M., Dény P., Gordien E. (2017). Genetic diversity and worldwide distribution of the *deltavirus* genus: A study of 2,152 clinical strains. Hepatology.

[9] Rizzetto M., Ponzetto A., Forzani I. (1990). Hepatitis delta virus as a global health problem. Vaccine.

[10] Hughes S.A., Wedemeyer H., Harrison P.M. (2011). Hepatitis delta virus. Lancet.

[11] Barros L.M.F., Gomes-Gouvêa M.S., Pinho J.R.R., Alvarado-Mora M.V., Dos Santos A., Mendes-Corrêa M.C.J., Caldas A.J.M., Sousa M.T., Santos M.D.C., Ferreira A.S.P. (2011). Hepatitis Delta virus genotype 8 infection in Northeast Brazil: Inheritance from African slaves?. Virus Res..

[12] Santos M.D.C., Gomes-Gouvêa M.S., Nunes J.D.C., Barros L.M.F., Carrilho F.J., Ferreira A.D.S.P., Pinho J.R.R. (2016). The hepatitis delta genotype 8 in Northeast Brazil: The North Atlantic slave trade as the potential route for infection. Virus Res..

[13] Brasil, Ministério da Saúde (2018). Boletim epidemiológico das hepatites virais. Secretaria de Vigilância em Saúde, Ministério da Saúde.

[14] Gomes-Gouvêa M.S., Soares M.D.C.P., de Carvalho Mello I.M.V.G., Brito E.M.F., Moia L.D.J.M.P., Bensabath G., Nunes H.M., Carrilho F.J., Pinho J.R.R. (2008). Hepatitis D and B virus genotypes in chronically infected patients from the Eastern Amazon Basin. Acta Trop..

[15] Botelho-Souza L.F., Souza Vieira D., de Oliveira dos Santos A., Cunha Pereira A.V., Villalobos-Salcedo J.M. (2015). Characterization of the Genotypic Profile of Hepatitis Delta Virus: Isolation of HDV Genotype-1 in the Western Amazon Region of Brazil. Intervirology.

[16] Di Filippo Villa D., Cortes-Mancera F., Payares E., Montes N., de la Hoz F., Arbelaez M.P., Correa G., Navas M.C. (2015). Hepatitis D virus and hepatitis B virus infection in Amerindian communities of the Amazonas state, Colombia. Virol. J..

[17] Cicero M.F., Pena N.M., Santana L.C., Arnold R., Azevedo R.G., Leal É.D.S., Diaz R.S., Komninakis S.V. (2016). Is Hepatitis Delta infections important in Brazil?. BMC Infect. Dis..

[18] Botelho Souza L.F. (2018). Análise Genomica do Vírus da Hepatite Delta Isolado na Amazônia Ocidental. Tese (Doutorado em Biologia Experimental) - Programa de Pós-graduação em Biologia experimental da Fundação.

[19] Casey J.L., Brown T.L., Colan E.J., Wignall F.S., Gerin J.L. (1993). A genotype of hepatitis D virus that occurs in northern South America. Proc. Natl. Acad. Sci. USA..

[20] Viana S., Paraná R., Moreira R.C., Compri A.P., Macedo V. (2005). High prevalence of hepatitis B virus and hepatitis D virus in the western Brazilian Amazon. Am. J. Trop. Med. Hyg..

[21] Paraná R., Kay A., Molinet F., Viana S., Silva L.K., Salcedo J.M., Tavares-Neto J., Lobato C., Rios-Leite M., Matteoni L. (2006). HDV genotypes in the Western Brazilian Amazon region: A preliminary report. Am. J. Trop. Med. Hyg..

[22] Gomes-Gouvea M.S., Soares M.C.P., Bensabath G., de Carvalho-Mello I.M.V.G., Brito E.M.F., Souza O.S.C., Queiroz A.T.L., Carrilho F.J., Pinho J.R.R. (2009). Hepatitis B virus and hepatitis delta virus genotypes in outbreaks of fulminant hepatitis (Labrea black fever) in the western Brazilian Amazon region. J. Gen. Virol..

[23] Rizzetto M., Canese M.G., Aricò S., Crivelli O., Trepo C., Bonino F., Verme G. (1977). Immunofluorescence detection of new antigen-antibody system (delta/anti-delta) associated to hepatitis B virus in liver and in serum of HBsAg carriers. Gut.

[24] Alvarado-Mora M.V., Romano C.M., Gomes-Gouvêa M.S., Gutierrez M.F., Carrilho F.J., Pinho J.R.R. (2011). Dynamics of Hepatitis D (delta) virus genotype 3 in the Amazon region of South America. Infect. Genet. Evol..

[25] Azarbahra M., Tajbakhsh E., Momtaz H. (2014). Phylogenetic analysis of hepatitis delta virus isolated from HBsAg positive patients in Shahrekord, Iran. Asian Pacific. J. Trop. Dis..

[26] Wu J.C., Chiang T.Y., Sheen I.J. (1998). Characterization and phylogenetic analysis of a novel hepatitis D virus strain discovered by restriction fragment length polymorphism analysis. J. Gen. Virol..

[27] Crispim M.A.E., Fraiji N.A., Campello S.C., Schriefer N.A., Stefani M.M.A., Kiesslich D. (2014). Molecular epidemiology of hepatitis B and hepatitis delta viruses circulating in the Western Amazon region, North Brazil. BMC Infect. Dis..

[28] Nakano T., Hadler S.C., Orito E., Shapiro C.N., Casey J.L., Mizokami M., Robertson B.H. (2001). Characterization of hepatitis D virus genotype III among Yucpa Indians in Venezuela. J. Gen. Virol..

[29] Marques V.A., Lewis-Ximenez L.L.S.R., Lampe E. Complete Genome Sequence of Hepatitis D Virus Isolated in Brazil. https://www.ncbi.nlm.nih.gov/nuccore/kc590319.1.

[30] Kumar S., Stecher G., Tamura K. (2016). MEGA7: Molecular Evolutionary Genetics Analysis Version 7.0 for Bigger Datasets. Mol. Biol. Evol..

[31] Rambaut A., Lam T.T., Max Carvalho L., Pybus O.G. (2016). Exploring the temporal structure of heterochronous sequences using TempEst (formerly Path-O-Gen). Virus Evol..

[32] Nogueira-Lima F.S. (2019). Oswaldo Cruz Foundation of Rondônia, Porto Velho, Rondônia. Complementary analysis with 55 HDV-1 sequences also obtained from Nucleotide database to compare temporal signal quality between different sequence sizes analyzed: Complete genome and R0 region.

[33] Hadler S.C., De Monzon M.A., Rivero D., Perez M., Bracho A., Fields H. (1992). Epidemiology and long-term consequences of hepatitis delta virus infection in the yucpa Indians of venezuela. Am. J. Epidemiol..

[34] Gill M.S., Lemey P., Faria N.R., Rambaut A., Shapiro B., Suchard M.A. (2013). Improving Bayesian population dynamics inference: A coalescent-based model for multiple loci. Mol. Biol. Evol..

[35] Rambaut A., Pybus O.G., Nelson M.I., Viboud C., Taubenberger J.K., Holmes E.C. (2008). The genomic and epidemiological dynamics of human influenza A virus. Nature.

[36] Ljunggren K.E., Patarroyo M.E., Engle R., Purcell R.H., Gerin J.L. (1985). Viral hepatitis in Colombia: A study of the hepatitis of the Sierra Nevada de Santa Marta. Hepatology.

[37] Buitrago B., Popper H., Hadler S.C., Thung S.N., Gerber M.A., Purcell R.H., Maynard J.E. (1986). Specific histologic features of Santa Marta hepatitis: A severe form of hepatitis delta-virus infection in northern South America. Hepatology.

[38] Bensabath G. (1983). Presença do Agente Delta associado-VHB em residentes do Município de Boca do Acre, micro região do Purus, Amazonas. Proceedings of the Nota Prévia. Programa E Resumos, XIX Congresso da Sociedade Brasileira de Medicina Tropical, Rio de Janeiro.

[39] Dias L.B., Coura J.R. (1985). Hepatite de Lábrea: Estudo de revisão em viscerotomias hepáticas dos anos de 1934 a 1940. Rev. Inst. Med. Trop. Sao Paulo.

[40] Fonseca J.C., Ferreira L.C., Brasil L.M., Castilho M.D.C., Moss R., Barone M. (1992). Fulminant Labrea hepatitis—The role of hepatitis A (HAV), B (HBV), C (HCV), and D (HDV) infection. (Preliminary report). Rev. Inst. Med. Trop. Sao Paulo.

[41] Fonseca J.C.F. (2010). da Histórico das hepatites virais. Rev. Soc. Bras. Med. Trop..

[42] Braga W.S.M., Melo H.O., Cossate M.D.B., Castilho M.C., Souza R.A.B., Brasil L.M., Fonseca J.C.F. (1998). Prevalência dos marcadores sorológicos dos vírus da hepatite B e Delta em população assintomática: Estudo do impacto do uso da vacina contra hepatite B em áreas hiperendêmicas, Itamarati-Amazonas, Vale do rio Juruá. Rev. da Soc. Bras. Med. Trop..

[43] Castilho M.C. (2012). Aspectos Epidemiológicos e da Biologia Molecular da Hepatite B em três Comunidades da Amazônia Ocidental Brasileira. Ph.D. Thesis.

[44] Scarponi C.F.D.O., Silva R.D.N.D., Souza Filho J.A.D., Guerra M.R.L., Pedrosa M.A.F., Mol M.P.G. (2019). Hepatitis Delta Prevalence in South America: A Systematic Review and Meta-Analysis. Rev. Soc. Bras. Med. Trop..

